# Quality of life of the SQ house dust mite sublingual immunotherapy tablet in Italian adolescents with house dust mite-induced allergic rhinitis

**DOI:** 10.1186/s13052-025-01947-3

**Published:** 2025-05-23

**Authors:** Francesco Catamerò, Mattia Giovannini, Simona Barni, Giulia Liccioli, Lucrezia Sarti, Leonardo Tomei, Benedetta Pessina, Claudia Valleriani, Chiara Marzi, Michela Baccini, Diego Peroni, Francesca Mori

**Affiliations:** 1 Allergy Unit, Meyer Children’s Hospital IRCCS, Florence, Italy; 2 Department of Health Sciences, University of Florence, Florence, Italy; 3 Immunology Laboratory, Meyer Children’s Hospital IRCCS, Florence, Italy; 4 Department of Statistics, Computer Science and Applications “G. Parenti, ” University of Florence, Florence, Italy; 5 Department of Clinical and Experimental Medicine, Section of Pediatrics, University of Pisa, Pisa, Italy

**Keywords:** Allergy, Adolescent, SLIT, Allergen immunotherapy, Quality of life

## Abstract

House dust mite (HDM) is the most common cause of perennial allergy worldwide, causing allergic rhinitis with or without conjunctivitis (AR/C). HDM-related clinical manifestations can be treated with allergy pharmacotherapy or allergen immunotherapy (AIT) in selected cases. AIT is acknowledged as the only therapeutic approach capable of modifying the course of allergic diseases. SQ HDM-SLIT tablets (Accarizax; Merck & Co, Kenilworth, NJ/ALK-Abellò, Hørsholm, Denmark) ensures a constant potency ratio of major HDM allergens of the *Dermatophagoides pteronyssinus* (DERM_PT) and *Dermatophagoides farinae* (DERM_FA) HDM species and has demonstrated beneficial effects on allergic rhinoconjunctivitis outcomes. In Italy, rhinoconjunctivitis affects up to as many as 40% of adolescents, but no studies on HDM AIT quality of life (QoL) regarding this exclusive cohort of patients have been specifically conducted. The aim of this study is to evaluate SQ HDM SLIT tablets’ performance in improving QoL in Italian adolescents. To assess the treatment at T0, T1, and T2, we employed the Rhinoconjunctivitis Quality of Life Questionnaire (RQLQ(s)), a standardized self-administered questionnaire designed for Italian patients aged 12 and above. RQLQ(s) median values at T2 and T1 were significantly lower than those at T0 (*p*-values equal to 0.0018 and 0.0051 for T0 vs. T2 and T0 vs. T1, respectively). Our findings suggest that the QoL of SQ HDM SLIT tablet is highly promising, demonstrating substantial potential in alleviating signs and symptoms. Our data suggest that QoL significantly improved with SQ-HDM SLIT, highlighting the potential importance of introducing this therapy for selected cases.

**To the Editor,**

House dust mite (HDM) is the most common cause of perennial allergy worldwide, causing allergic rhinitis with or without conjunctivitis (AR/C). HDM-related clinical manifestations can be treated with allergy pharmacotherapy or allergen immunotherapy (AIT) in selected cases [1].

AIT, with the subcutaneous (SCIT) or sublingual (SLIT) administration of the culprit allergen is acknowledged as the only therapeutic approach capable of modifying the course of allergic diseases thereby reducing signs and symptoms and offering long-term post-treatment benefits [2, 3].

SQ HDM-SLIT tablets (Accarizax; Merck & Co, Kenilworth, NJ/ALK-Abellò, Hørsholm, Denmark) ensure a constant potency ratio of major HDM allergens of the *Dermatophagoides pteronyssinus* (DERM_PT) and *Dermatophagoides farinae* (DERM_FA) HDM species and has demonstrated beneficial effects on allergic rhinoconjunctivitis outcomes in European, Japanese and North American trials [4–6]. SQ HDM SLIT-tablets are fast-dissolving (< 10 s) freeze-dried tablet with a 1:1 mixture of allergen extracts from the HDM species DERM_PT and DERM_FA. The source material of DERM_PT and DERM_FA, containing extracted mite bodies, as well as mite feces, provides the tablet with the broadest possible spectrum of major and minor allergens. In addition, a highly standardized production process ensures a 1:1:1:1 potency ratio of the major allergens Der p 1, Der f 1, Der p 2, and Der f 2. The 12 SQ-HDM dose contains roughly 15 mg of group 1 mite allergens (Der f 1 and Der p 1 combined) and 15 mg of group 2 mite allergens (Der f 2 and Der p 2 combined).

In Italy, rhinoconjunctivitis affects up to as many as 40% of adolescents [7], but no studies on HDM AIT quality of life (QoL) in this exclusive cohort of patients have been conducted. The aim of this study is to evaluate SQ HDM SLIT tablets’ performance in improving QoL in Italian adolescents.

The eligibibility for the study was determined following the inclusion and exclusion criteria outlined in the EAACI guidelines [8]. Skin prick tests (SPTs) were performed on all children using commercial allergen extract (Lofarma, Milan, Italy). The SPTs were considered positive if the weal diameter was equal to or greater than 3 mm at 15 min-reading. The positive and negative controls for the SPTs were obtained using histamine (10 mg/ml; Lofarma, Milan, Italy) and normal saline, respectively. The specific IgE (sIgE) were determined using a commercial assay (ImmunoCAP system-Thermo FisherScientific, Uppsala, Sweden) with a positive cut-off point set at 0.1 kUA/L. Written informed consent was obtained from the children's parents for all procedures performed.

We collected data from 15 Italian adolescents from November 2021 to December 2023. Subjects received daily SQ HDM SLIT tablets for up to approximately 52 weeks. First administration of treatment occurred to the day-hospital site of Allergy Unit of Meyer Children's Hospital IRCCS, followed by a 30-min observation under medical supervision, to educate patients on the proper tablet assumption and to monitor immediate first assumption-related signs and symptoms. After treatment tolerance had been confirmed, subsequent doses were self-administered at home. Patients were clinically evaluated at baseline (T0), at about six months after the beginning of AIT (T1) and about one year after the beginning of AIT (T2). Compliance was monitored during clinical visits.

To assess the treatment at T0, T1, and T2, we employed the Rhinoconjunctivitis Quality of Life Questionnaire (RQLQ(s)), a standardized self-administered questionnaire designed for Italian patients aged 12 and above.

The clinical assessment includes evaluating sneezing, watery nasal discharge, nasal itching, nasal congestion, sleep disturbances, difficulties at work or school, and impairment of daily activities. SPTs were performed at T0 and T2. sIgE against DERM_PT and DERM_FA, molecular analysis for Der p 1, Der p 2, Der p 10 and Der p 23, and total IgE were dosed at T0 and then repeated at T2.

We compared the clinically assessed binary variables over the three time-points through the non-parametric Cochran’s Q-test, followed by pair-wise McNemar’s tests. We performed two-sided Wilcoxon Signed Rank test to evaluate differences in SPT, sIgE and molecular analysis over the two time-points (T0 and T2). The treatment, measured by the RQLQ(s), was assessed through a Friedman Rank Sum test, followed by pair-wise Wilcoxon Signed Rank tests over the three time-points. A multiple linear model was constructed to explore the relationship between age, sex, family history of atopy, comorbidities and RQLS(s) at T0 at T2. We set the significance level at 0.05, and we applied the Bonferroni correction to all the multiple comparisons and post-hoc tests. The statistical analyses were performed on R Studio v. 4.3.1. (Posit Software PBC, United States of America).

Data from 15 patients (11 males, 73.3%) under 18 years of age (median age 14 years, IQR = [13.0, 15.0 years]) were retrospectively retrieved. Details of their demographic and clinical characteristics are shown in Table 1. One patient, not included in the analysis, manifested poor compliance and discontinued the treatment after six months.

**Table 1:** Demographic and clinical characteristics of the population

Baseline characteristics	Total Patients (*n*=15)
Median age (IQR) years	14 (13 – 15)
Male sex (%)	11 (73.3)
Median SPT DERM_PT (IQR) mm	7 (6 – 10)
Median SPT DERM_FA (IQR) mm	7 (6 – 10)
Total IgE (IQR) kU/L	279 (115 – 1079)
Sensitization to other inhalants (%)	9 (60.0)
Family history of atopy (%)	9 (60.0)
Other atopic conditions (%)	6 (40.0)
Clinical manifestations at baseline	
Sneezing (%)	14 (93.3)
Watery rhinorrhea (%)	14 (93.3)
Nasal itching (%)	13 (86.7)
Nasal obstruction (%)	12 (80.0)
Sleeping disorders (%)	3 (20.0)
Difficulties at school (%)	4 (26.7)
Daily activities impairment (%)	3 (20.0)

*DERM_PT* *Dermatophagoides pteronyssinus, DERM_FA* *Dermatophagoides farinae,* *IQR* interquartile range, *SPT* Skin Prick Test

RQLQ(s) median values at T2 (0.8570, IQR = [0.2855, 1.240]) and T1 (1.1070, IQR = [0.5445, 1.5172]) were significantly lower when compared to the median value at T0 (2.042, IQR = [1.268, 2.893]), with Bonferroni-corrected *p*-values equal to 0.0018 and 0.0051 for T0 vs. T2 and T0 vs. T1, respectively (Fig. 1). No statistically significant associations between RQLQ(s) values at T2 and age, sex, family history of atopy, and comorbidities at T0 were found.

**Fig. 1:**
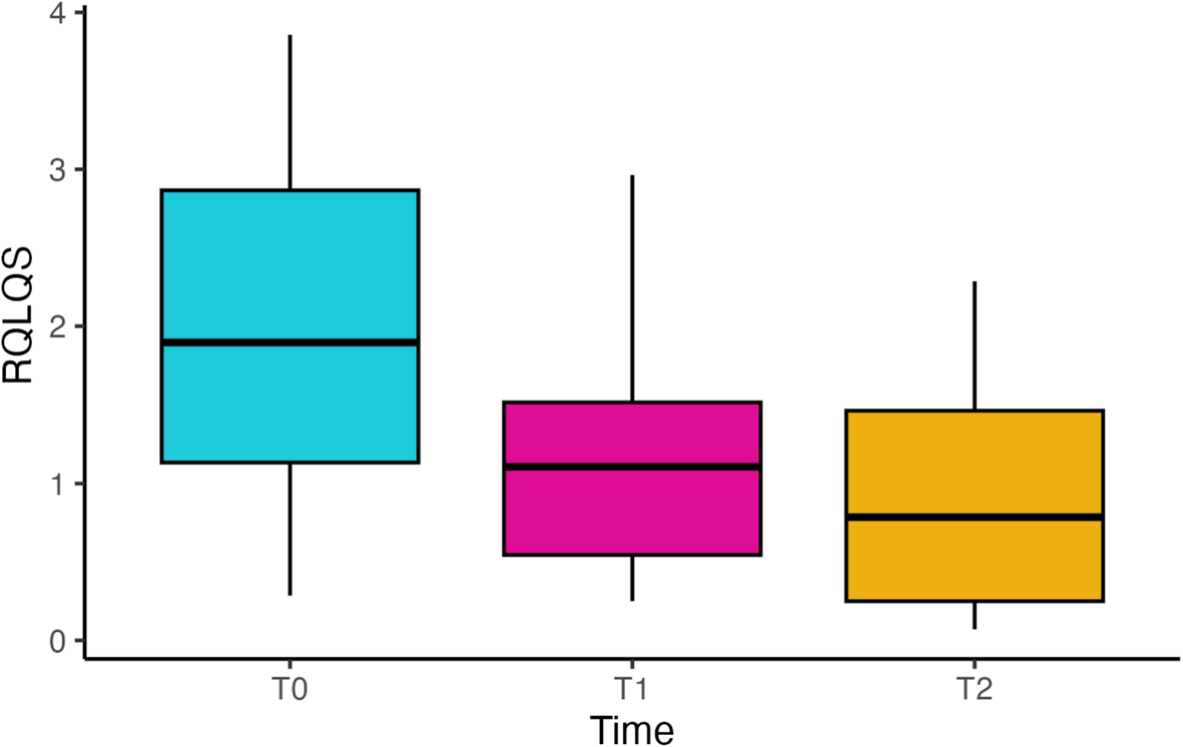
Box plot of RQLQ(s) values obtained at T0, T1 and T2

Sneezing (*p*-value = 0.039), watery rhinorrhea (*p*-value = 0.023), and nasal itching (*p*-value = 0.023) showed considerable improvement across the three time points. No differences were found for all the other sign and symptoms (Fig. 2).

**Fig. 2:**
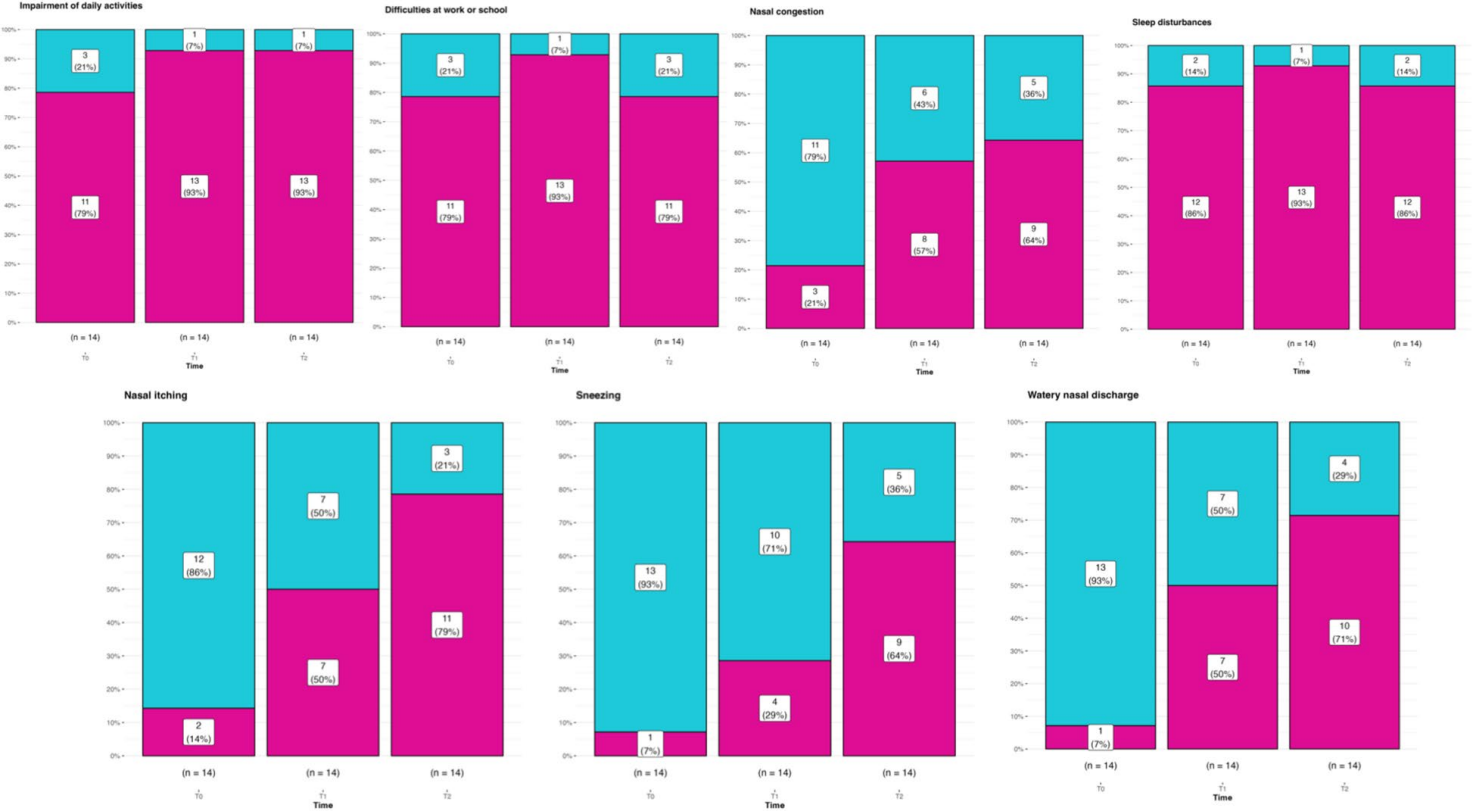
T0, T1 and T2 clinical assessment evaluating sneezing, watery nasal discharge, nasal itching, nasal congestion, sleep disturbances, difficulties at work or school, and impairment of daily activities

Median values of sIgE against DERM_PT (38.80, IQR = [17.45, 69.90]) and DERM_FA (26.10, IQR = [12.82, 40.15]) at T0 were significantly lower than those at T2 (DERM_PT = 84.60, IQR = [40.25, 101], *p*-value = 0.03; DERM_FA = 59.50, IQR = [38.05, 101], *p*-value = 0.01). Molecular analysis highlighted a statistically significant difference between T0 and T2: Der p 1 (20.70, IQR = [6.49, 34.40] at T0; 62.40, IQR = [34.00, 82.80] at T2; Bonferroni-corrected *p*-value = 0.00005) and Der p 23 (8.78, IQR = [2.27, 15.90] at T0; 26.60, IQR = [6.24, 36.25] at T2; Bonferroni-corrected *p*-value = 0.03) (Fig. 3).

**Fig. 3:**
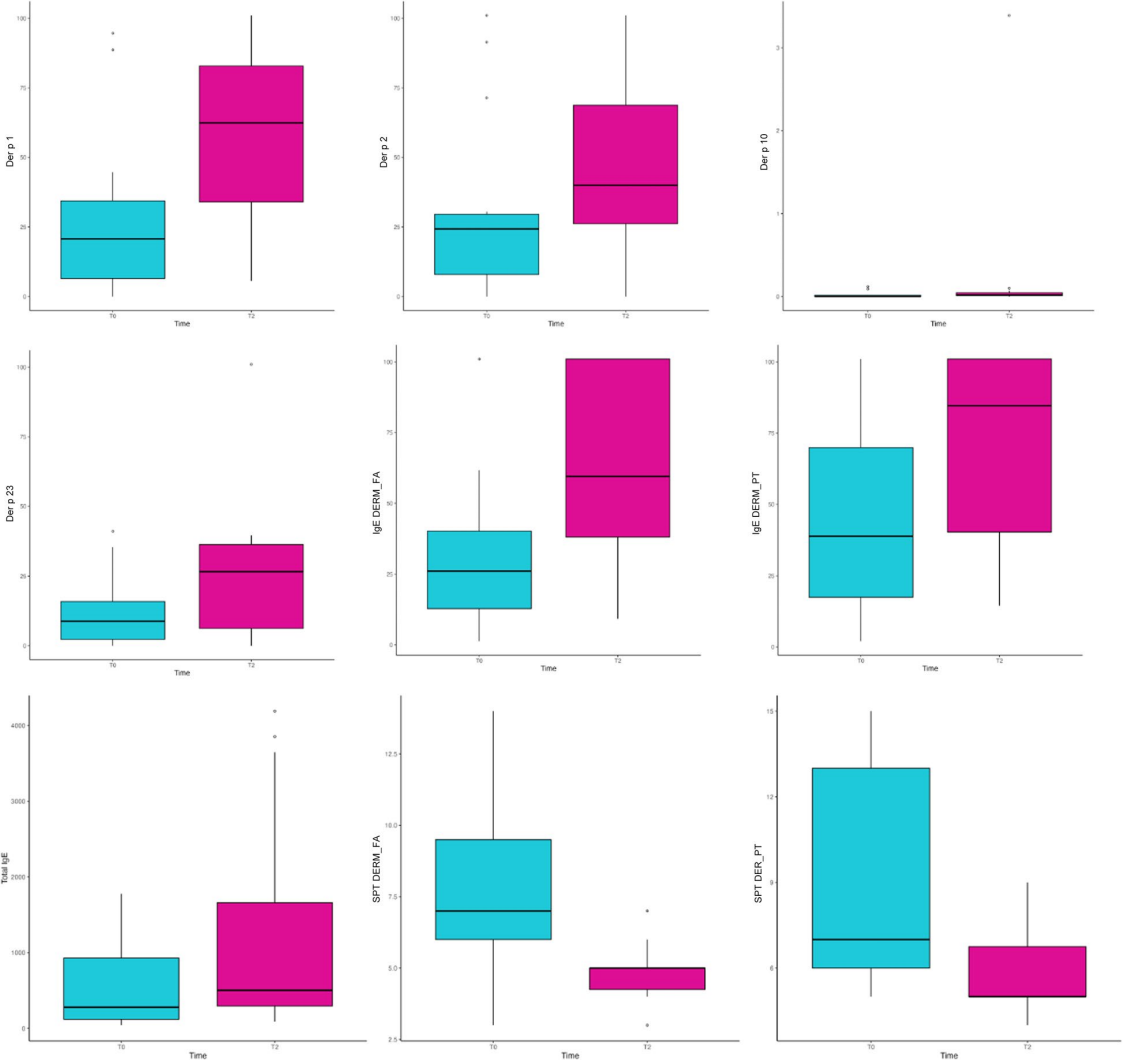
Box plot of total IgE, sIgE values against *Dermatophagoides pteronyssinus (*DERM_PT)*; Dermatophagoides farinae (*DERM_FA); molecular analysis and skin prick test (SPT) against DERM_PT and DERM_FA at T0 and T2

In terms of adverse events, three patients referred mouth itching that resolved spontaneously after about 15 days of treatment. No systemic reactions were observed.

Our study aimed at evaluating the QoL of SQ HDM SLIT tablet in Italian adolescents with HDM-induced allergic rhinoconjunctivitis, this way marking a significant contribution to the understanding of allergy treatment in this specific population. Our findings suggest that the QoL of SQ HDM SLIT tablet is highly promising, demonstrating remarkable potential in alleviating signs and symptoms. Our data also suggest that QoL significantly improved with SQ HDM SLIT, which highlights the prospective importance of introducing this therapy for selected cases.

Notably, our research indicates that the use of this medication is associated with efficacy and minimal to no adverse effects, highlighting the safety profile of the treatment option. Furthermore, the implications of utilizing SQ HDM SLIT tablet extend beyond efficacy and safety profile. We observed a marked improvement in nasal clinical manifestations, consistent with findings reported in literature [9]. The ability to mitigate signs and symptoms such as sneezing, watery rhinorrhea, and nasal itching can alleviate the physical discomfort and functional limitations experienced by patients, enabling them to engage more in their personal lives.

One limitation of our study, which may reduce the ability to detect statistically significant differences, is the sample size. Consequently, our findings reccomend further investigation in larger cohorts or through comparison with data from other Italian centers, which, to the best of our knowledge, have not yet been analyzed in the current literature. In particular, the lack of statistically significant associations between RQLQ(s) scores at T2 and variables such as age, sex, family history of atopy, and comorbidities at T0 may be attributable to the sample size.

Overall, our findings underscore the role of SQ HDM SLIT tablets in the management of allergic rhinitis among Italian adolescents with HDM-induced allergic rhinitis, emphasizing its role to improve patient outcomes and enhance overall well-being. Further clinical exploration would be beneficial to fully elucidate this treatment's long-term efficacy and benefits.

## Data Availability

Aggregate analyses are available on reasonable request to the corresponding author.

## Abbreviations

HDM: House dust mite
AR/C: Allergic rhinitis with or without conjunctivitis
AIT: Allergen immunotherapy
SCIT: Subcutaneous immunotherapy
SLIT: Sublingual immunotherapy
DERM_PT: *Dermatophagoides pteronyssinus*
DERM_FA: *Dermatophagoides farinae*
QoL: Quality of life
SPTs: Skin prick tests
sIgE: Specific IgE
RQLQ(s): Rhinoconjunctivitis Quality of Life Questionnaire
